# Niosomal Hydrogel Loaded With Bromelain: A Promising Solution for Reducing Skin Collagen in Scleroderma Patients

**DOI:** 10.1111/jcmm.70478

**Published:** 2025-07-24

**Authors:** Hanieh Ardeshiri, Mina Ghorbani, Mohammad Ali Nazarinia, Kimia Falamarzi, Ali Mohammad Tamaddon, Negar Azarpira

**Affiliations:** ^1^ Department of Medical Nanotechnology School of Advanced Medical Sciences and Technologies Shiraz Iran; ^2^ Chemical Engineering Department University of Waterloo Waterloo Ontario Canada; ^3^ Department of Internal Medicine, School of Medicine Shiraz University of Medical Sciences Shiraz Iran; ^4^ Student Research Committee Shiraz University of Medical Sciences Shiraz Iran; ^5^ Department of Pharmaceutics, School of Pharmacy Shiraz University of Medical Sciences Shiraz Iran; ^6^ Transplant Research Center Shiraz University of Medical Sciences Shiraz Iran

**Keywords:** bromelain, drug delivery, fibrosis, nanoparticle, niosome, scleroderma

## Abstract

Skin fibrosis in scleroderma is a chronic and debilitating condition that affects the quality of life of patients. In this study, we fabricated a niosomal hydrogel containing bromelain to reduce skin collagen and improve skin softness in scleroderma patients. The niosomes were prepared using the thin film hydration method and exhibited a drug loading efficiency of 52.2%. They demonstrated high monodispersity, with mean sizes lower than 200 nm. The results showed a significant reduction in mean dermal thickness from 1195 μm before treatment to 750 μm after the 2‐month treatment period. This reduction in dermal thickness and improved skin softness can be attributed to the decomposition of accumulated skin collagen in individuals with scleroderma. Additionally, the treatment was found to be effective in reducing skin collagen levels compared to conventional treatment options that merely impede collagen synthesis. The current study underscores the potential of bromelain niosomal hydrogel as a promising strategy for managing skin fibrosis in scleroderma.

**Abbreviations**
ANA: anti‐nuclear antibody
anti‐Scl70: anti‐topoisomerase I
BBD: bromelain‐based debridement
dcSSc: diffuse cutaneous systemic sclerosis
DLS: dynamic light scattering
EE: encapsulation efficacy
lcSSc: limited cutaneous systemic sclerosis
MPA: Multi Probe Adapter System
mRSS: modified Rodnann skin score
NAC: N‐acetylcysteine
PAA: polyacrylic acid
PDI: polydispersity index
PG: prostaglandin
SEM: scanning electron microscope
SSc: systemic sclerosis

## 1 Introduction

Systemic sclerosis (SSc) is a rare chronic autoimmune disease characterised by vascular damage and excessive collagen production causing progressive fibrosis of the skin and internal organs [1, 2]. SSc affected 20 per 100,000 cases and is more prevalent among women [3]. Based on the extent of cutaneous involvement, SSc is divided into two main subtypes: limited cutaneous SSc (lcSSc) and diffuse cutaneous SSc (dcSSc) which can be life‐threatening due to its rapid progression and early involvement of internal organs [4]. Even though SSc is a multi‐systemic disease, skin involvement is the main and most frequent manifestation of SSc [5]. The Raynaud's phenomenon, cutaneous sclerosis, ulcers and calcification, telangiectasia, and skin hyper‐ or hypopigmentation as well as itchy, painful and tightened skin are among the cutaneous presentations of SSc [5, 6]. Other complications are joint contracture, digital ulcer, sclerodactyly, vascular insufficiency and ischaemia resulting in disability and limitations in patients' daily activities [2]. Ischaemia reperfusion response, generation of profibrotic cytokines and oxidative stress are the main pathomechanisms of fibrosis in SSc, leading to fibroblasts transdifferentiation to myofibroblasts, collagen synthesis and ECM remodelling [7, 8]. The predominant strategy for treating fibrosis in patients has been centred on inhibiting inflammation associated with fibrosis through medications like corticosteroids, but the efficacy of this approach is limited [9]. Numerous clinical trials have attempted to alleviate fibrosis by either diminishing oxidative stress (via agents like N‐acetylcysteine (NAC) [10] tocopherol (vitamin E) [11], or decreasing the expression of pro‐fibrotic cytokines or genes with treatments such as monoclonal antibodies against TGF‐β [12] or tyrosine kinase inhibitor imatinib [13] However, these treatments did not provide a solution for excess collagen in the skin; instead, they contributed to a reduction in collagen production.

Despite systemic and localised therapeutic strategies suggested for SSc skin involvement, its management remains challenging since there are still no effective disease‐modifying medications [14, 15]. Therefore, finding a safe and efficient option for SSc skin fibrosis is essential. In our pursuit of finding a solution to reduce skin collagen in patients with scleroderma, we came across an enzyme known as bromelain, which demonstrates proteolytic activity. Bromelain, the main and most widespread proteolytic enzyme found in pineapple ( *Ananas comosus* ), is naturally present in plants of the Bromeliaceae family. These enzymes belong to a group of endopeptidases that are specific to the tissues of this plant family. Bromelain, primarily found in the stem and fruit of the pineapple plant, exhibits proteolytic and fibrinolytic properties [16, 17, 18, 19]. These groups of enzymes are generally regarded as safe materials and can be consumed with daily doses ranging from 200 to 2000 mg/kg over an extended period without harmful effects [20]. Previous studies reported numerous biological functions of bromelain including inflammatory response modulation, suppressing platelet aggregation, fibrinolysis, as well as anti‐carcinogenic and antibacterial properties, suggesting its therapeutic utilisation in a variety of diseases such as osteoarthritis, rheumatoid arthritis, cancers, cardiovascular diseases, skin wounds and burns with no serious side effects [21, 22, 23].

Bromelain‐based debridement (BBD) is a quick, effective and secure method for enzymatic debridement in burn injuries. This process uses a high‐concentration solution of proteolytic enzymes and offers the advantage of selectivity by removing non‐viable tissue while preserving the viable tissue. In vivo studies have demonstrated that debridement with this agent effectively eliminates eschar without causing damage to viable tissue, leading to a clean wound bed with viable dermis or subdermal tissue [24, 25, 26, 27, 28, 29, 30, 31]. Moreover, many articles reported the effectiveness of bromelain enzyme in hydrolysing collagen in the pharmaceutical, cosmetic and food industries [32, 33, 34].

Nanocarriers are utilised to protect drugs from enzymatic breakdown and biological environments within the body, facilitate targeted delivery of drugs to specific cells and areas, and enhance their ability to penetrate biological barriers. Various types of nanocarriers are available, including liposomes, solid lipid nanoparticles, niosomes, porous nanoparticles, polymer nanoparticles and many more [35]. Niosomes are a type of vesicular nanocarrier composed of non‐ionic surfactants and cholesterol and are known for their stability, affordability, ease of manufacturing and superior drug delivery properties. They share similarities with surfactants, making them capable of easily penetrating the skin. Niosomes are considered an effective drug delivery system due to their unique amphiphilic properties [36].

Several recent studies have demonstrated the potential of niosomal systems and nanocarriers in enhancing topical drug delivery, particularly in dermatological conditions where skin barrier dysfunction poses a challenge. Niosomes, as non‐ionic surfactant‐based vesicles, have been widely reported to improve drug encapsulation efficiency, stabilityand skin penetration [37, 38, 39, 40, 41]. The thin‐film hydration method, which was employed in our study, has been extensively used for the preparation of optimised niosomal formulations, ensuring controlled size distribution and high drug entrapment efficiency [39, 40].

Furthermore, studies have highlighted the role of cholesterol and surfactant ratios in determining the physicochemical properties of niosomes. The selection of surfactants such as Span 60 and Tween 80 has been shown to influence vesicle size, polydispersity index (PDI) and drug retention within the stratum corneum and viable epidermis, leading to enhanced drug deposition and therapeutic efficacy [38, 40, 41].

Ex vivo permeation studies have consistently demonstrated that niosomal formulations significantly enhance drug penetration through the skin while simultaneously increasing drug deposition in deeper epidermal layers, thereby ensuring localised therapeutic effects [37, 39]. These findings align with our study's objective of improving bromelain delivery in scleroderma patients, as enhanced dermal retention is crucial for collagen degradation and therapeutic efficacy. Additionally, in vivo studies on psoriasis models have shown that niosomal formulations not only improve drug permeation but also lead to superior clinical outcomes compared to conventional formulations, further supporting the rationale behind our approach [38, 40].

Incorporating these insights, we have revised the formulation section to align with the latest advancements in niosomal drug delivery systems. We appreciate the reviewer's valuable suggestions, which have helped enhance the scientific rigor of our study.

Given the increased skin collagen and stiffness in scleroderma, we hypothesized that the application of bromelain enzyme could potentially reduce collagen content in the skin of patients. To enhance the enzyme's efficiency in reaching the dermis, we encapsulated bromelain in niosomes. For topical application, we incorporated these niosomes into a hydrogel. Finally, we conducted a case study to evaluate its effectiveness.

## 2 Materials and Methods

### 2.1 Materials

Span 60 and polyacrylic acid were purchased from (Alfa Aesar, USA); cholesterol, triethanolamine, soy lecithin, methanol, chloroform and bromelain were purchased from (Merk, Germany).

### 2.2 Methods

#### 2.2.1 Niosomal Formulation

To prepare, a mixture of Span 60, soy lecithin and cholesterol was dissolved in a combination of chloroform and methanol at a mass ratio of 1:1:0.5, and the resulting mixture was placed in a round‐bottom flask. The solvent was then removed by rotary evaporation at 180 rpm and 60°C for 30 min, leaving a thin layer on the surface of the flask. The flask was then placed in a desiccator for 24 h to eliminate any residual solvent. The niosomes were hydrated by 20 mL of water with a 6 mg/mL concentration of bromelain for 3 h in a water bath (Bandelin UW 2070, Germany) without heating, followed by sonication under an ice bath for 30 min, with 1‐min intervals of sonication and rest.

#### 2.2.2 Preparation of Hydrogels

To facilitate applying niosomes onto the skin, a polyacrylic acid (PAA) hydrogel was used. A 1.0% PAA was dispersed in deionised water, and the pH was adjusted to about 6.0 using triethanolamine. The mixture was left overnight to allow the PAA chains to fully expand, after which it was combined with the niosome suspension. The concentration of bromelain in the niosomal hydrogel was ultimately adjusted to be 2 mg/mL.

#### 2.2.3 Characterisation

##### 2.2.3.1 Morphological Analysis of Niosomes

To evaluate the morphology of the niosomes, a scanning electron microscope (SEM) (Xl30‐Philips‐America) with a voltage of 16 kV was employed. The sample was first dried and then coated with a 2–3 nm gold layer thickness using a sputter coating device.

##### 2.2.3.2 Niosome Size Determination

The dynamic light scattering (DLS) instrument (Horiba, SZ‐100, Japan) was employed to assess the size distribution (polydispersity index, PDI), vesicle size (nm), and zeta potential (mV) of the niosomes. To perform the analysis, the samples were appropriately diluted 10 times with distilled water and measured at a temperature of 25°C and an angle of 90°. Additionally, the zeta potential of the prepared samples was determined in deionised water at 25°C.

##### 2.2.3.3 FT‐IR

The FTIR test identifies the functional groups of the compounds. To evaluate any chemical interaction between ingredients of niosome and drug on the empty niosomes, niosomes with drugs and pure drugs, Fourier Transform Infrared (FT‐IR) analyses were performed (BRUKER instrument, Germany). Tablets were formed by mixing KBr pellets with these compounds, and FTIR spectra were recorded over the range of 4000–400 cm^−1^.

##### 2.2.3.4 Encapsulation Efficacy (EE)

The drug loaded in the niosomes was specified by centrifuging the drug‐containing niosomes using an ultracentrifuge machine (3‐ck sigma) at 20,000 rpm for 1 h to separate the drug‐containing niosomes and free drug. A sample was taken from the supernatant, which contained the free drug, and the concentration of the free drug was measured using a nanodrop device (Thermo Scientific). The drug load was calculated using the following formula:

$$\mathrm{EE}\left(\%\right)=\left(\frac{E_{1}-E_{2}}{E_{1}}\right)\times 100$$

where *E*_1_ is the amount of drug added and *E*_2_ is the amount of free drug.

#### 2.2.4 In Vitro Release Profile of Bromelain

Following the removal of the non‐encapsulated bromelain, 5 mL of each formulation loaded with bromelain was individually placed in a tube with a molecular weight cutoff of 14 kDa. The dialysis tubes were then submerged in 50 cc DI water and stirred continuously at 37°C. Samples were taken at predetermined time points up to 28 h. At each sampling time point, 300 μL of the dialysis media from each sample was extracted and replaced with fresh 300 μL of water preheated to 37°C to maintain sink conditions. The concentration of bromelain in the samples was measured using nanodrop at each time point. The experiments were performed in triplicate, and the mean ± SD is reported.

#### 2.2.5 Study Design

##### 2.2.5.1 Ethical Considerations

This is a single‐case study conducted on a patient diagnosed with diffuse cutaneous systemic sclerosis. Current study protocols were approved by the Ethics Committee of Shiraz University of Medical Sciences (Approval code: IR.SUMS.REC.1400.870) and written informed consent was obtained from the participant before enrolment in the study.

##### 2.2.5.2 Case Report

One of the authors of this article, who also participated in this research, is a 28‐year‐old female who was diagnosed with (dcSSC) 15 years ago. Her diagnosis is characterised by sclerodactyly, arthralgia, positive anti‐nuclear antibody (ANA) and anti‐topoisomerase I (anti‐Scl70). At the time of this study, the patient exhibited clinical manifestations including diffuse skin tightening, telangiectasia, Raynaud's phenomenon and gastroesophageal reflux disease [42]. Her current medication regimen included azathioprine, prednisolone, diltiazem, omeprazole and domperidone, with medical assessments and health condition monitored by a rheumatologist.

##### 2.2.5.3 Interventions

One gram of hydrogel containing 2 mg of bromelain was applied to the palmar and dorsal surfaces of the participant's hand daily for 2 months.

##### 2.2.5.4 Outcome Measures

1. Skin thickness was evaluated using the modified Rodnan Skin Score (mRSS), a validated test for measuring skin thickness. A rheumatologist performed these measurements on the bromelain‐applied areas before and after the intervention. The mRSS was calculated by gently pinching the skin between the index finger and thumb and assigning scores: 0 for normal skin with fine wrinkles and no thickness, 1 for mild skin thickness (skin folds between the examiner's fingers), 2 for moderate skin thickness with difficulty in skin folding and no wrinkles, and 3 for severe skin thickness without the ability to fold skin between examiner's fingers [43, 44].
2. Epidermal and dermal thickness of the five selected areas was measured using skin ultrasonography with the C + K Multi Probe Adapter System (MPA, Germany) before and after the intervention.
3. Patients self‐reported outcomes were assessed and observed by a rheumatologist before and after the intervention to evaluate the skin‐related parameters and functions such as skin tightness, thickness, pain and itching.

#### 2.2.6 Statistical Analysis

All data were presented as mean ± standard deviation. The comparison between skin thickness before and after treatment was recorded and analysed using the Mann–Whitney *U* test by SPSS software (version 26). *p* value < 0.05 was considered statistically significant to determine any substantial changes in the thickness due to the treatment.

## 3 Results

### 3.1 Characterisation of Niosomes

**FIGURE 1:**
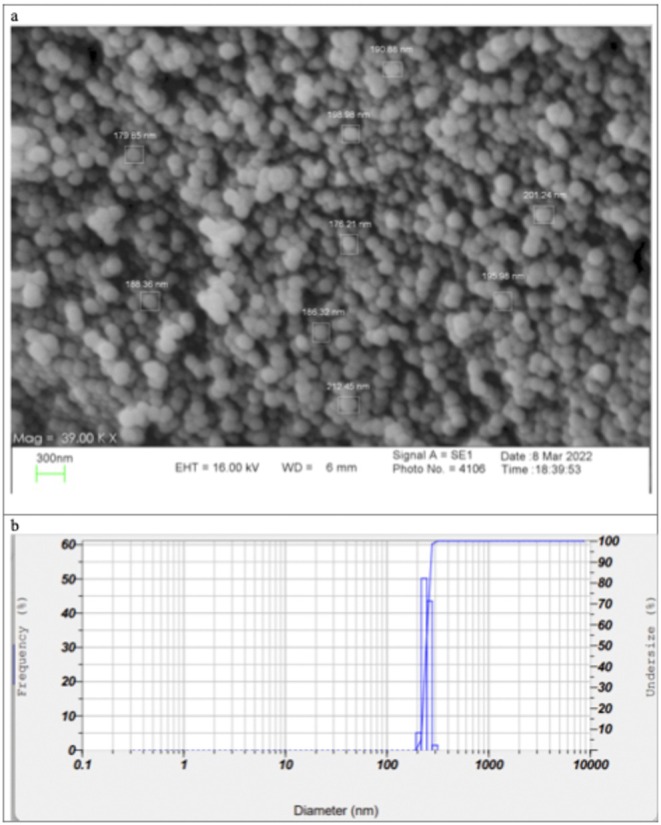
(a) SEM images of Bromelain niosomes, (b) Size distribution graph in terms of the frequency of bromelain niosomes mean size of 243.9 and with PI 0.33.

The SEM images, as shown in Figure 1a, indicate that the niosomes exhibit a spherical shape and high monodispersity, with a size of lower than 200 nm. However, the DLS analysis (Figure 1b.) results revealed that the size of the bromelain niosomes was 243.9 nm (PI = 0.330), with a zeta potential of −44.1 mV. Moreover, the encapsulation efficieny of bromelain in niosomes was estimated to be 52.2% ± 1.74.

**FIGURE 2:**
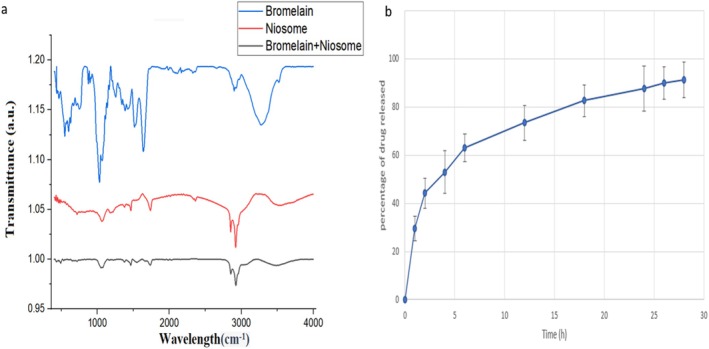
(a) FTIR spectra of empty niosomes, bromelain, and bromelain‐loaded niosomes. (b) The percentage of bromelain released from the niosomes at 37°C over a period of 28 h.

In the FTIR analysis of drug‐loaded niosomes, two points are important. After niosome formation, a broad peak in the range of 3000–3700 cm^−1^ is observed indicating a strong hydrogen bond between cholesterol and Span 60 [43, 44]. Secondly, when drug‐containing niosomes are formed, there should be no appearance of a new peak in the FTIR spectrum along with the peaks of pure drug and drug‐free niosomes, since the appearance of a new peak would suggest a chemical reaction between the drug and niosome components. Bromelain niosomes meet these criteria. Also, the drug peaks are observed with lower intensities in the niosome formulation, indicating the drug loading in the niosomes. These FTIR spectra are shown in Figure 2a.

Furthermore, a release study was conducted to investigate the bromelain release profile of the niosomal hydrogel. Figure 2b demonstrates that the niosomal hydrogel formulation exhibits a biphasic pattern of drug release. There is an initial rapid release phase in which approximately 60% of the drug is released within the first 8 h, followed by a sustained release phase over the next 20 h, during which the drug is released at a slower rate.

### 3.2 Modified Rodnann Skin Score (mRSS)

Evaluation of skin thickness using mRSS before and after the intervention by the same examiner demonstrated a decrease in mean score from 3 to 1 in bromelain‐applied areas.

### 3.3 Ultrasound Imaging of Cutaneous Lesions

**FIGURE 3:**
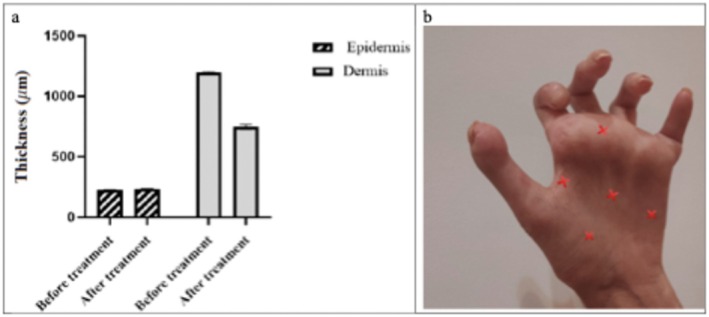
(a) The thickness of the epidermis and dermis was measured using skin ultrasound before and after 2 months of treatment, (b) Selected areas of the patient's hand underwent skin ultrasound evaluation to measure epidermal and dermal thickness.

To evaluate the effectiveness of treatment on skin thickness, skin ultrasound was performed before and after the intervention on bromelain niosomal hydrogel‐applied areas. Our results showed that there was an increase in the average thickness of the epidermis from mean rank of 3 before treatment to mean rank of 8 after treatment (*p*‐value = 0.008). Additionally, the thickness of the dermis significantly decreased from mean rank of 8 before treatment to mean rank of 3 after treatment (*p*‐value = 0.008). The thickness of the dermis changed from a numerical average of 1195 μm before treatment to 750 μm after treatment (Figure 3a.). The selected areas where epidermal and dermal thickness were measured are demonstrated in Figure 3b.

**FIGURE 4:**
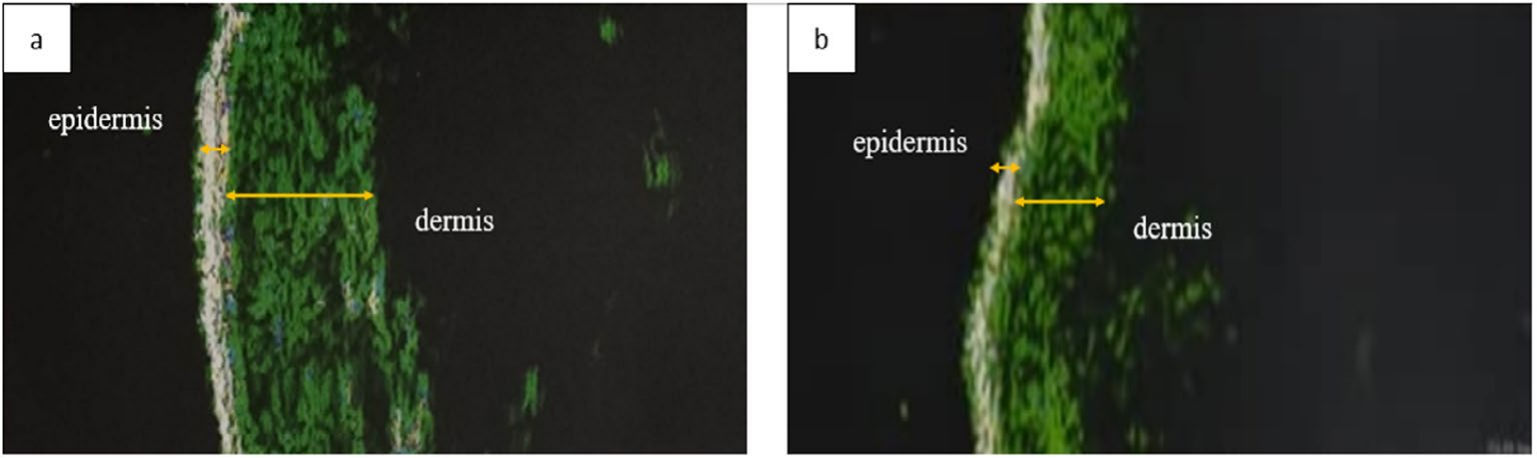
Ultrasound imaging of cutaneous lesions (a) before treatment (b) after treatment.

Table 1 presents the quantitative results, while Figure 4 illustrates the changes in skin thickness.

**TABLE 1:** The results of skin ultrasound imaging of selected areas.

	Before treatment	After treatment	*p*
Numeric average of epidermis thickness (μm)^a^	227	234	—
Numeric average of dermis thickness (μm)^a^	1196	745	—
Average of epidermis thickness	3	8	0.008
Average of dermis thickness	8	3	0.008

^a^ It is a descriptive statistics.

### 3.4 Patient's Self‐Reported Outcomes

The participant's self‐reported outcomes are described in Table 2. Ten days after treatment initiation, the participant reported a decrease in skin tightness and dryness with no sign of erythema and itching. After 2 months, the participant declared a significant improvement in the natural brightness and softness of her skin. Additionally, she reported a reduction in pain during blood sampling and easier locating of the vein; although, she did not describe any improvements in the flexion contracture of the fingers. No adverse effects such as pain, oedema, rash, erythema or itching were reported.

**TABLE 2:** Participant's self‐reported outcomes.

Outcomes	Before the intervention	After the intervention
Skin tightness	Severe	Moderate
Skin dryness	Severe	Moderate
Experiencing pain during blood sampling	Severe	Moderate
Itching	—	—
Interfered with daily activity	Severe	Severe
Difficulty in localising vein	Severe	Moderate
Skin discoloration	Severe	Moderate

*Note:* Outcomes range from mild, moderate to severe.

## 4 Discussion

SSc, a chronic autoimmune disease, is characterised by an abnormal immune response, vasculopathy and excessive fibrosis of the skin and various internal organs [45]. Despite being a multi‐systemic disease, skin involvement is one of the main features of scleroderma that affects patients' quality of life considerably [4]. Immunosuppressive agents (such as mycophenolate mofetil, azathioprine, methotrexate and cyclophosphamide), corticosteroids, biological agents and haematopoietic stem cell transplantation are among systemic therapies for SSc [15]. Moreover, topical corticosteroids and immunomodulators, as well as autologous fat grafting and phototherapy, are considered as local therapeutic measures for cutaneous involvement in SSc [46]. However, there is no FDA‐approved treatment for skin fibrosis in SSc, and current options lack significant efficiency. They are incapable of reversing the fibrosis process and also have side effects that limit their use [15, 47]. Hence, finding an alternative strategy is of great importance. Bromelain, a proteolytic enzyme with a variety of biological functions, might be a potential candidate for skin involvement in SSc [48]. The results of this study demonstrate that treatment with niosomal hydrogel containing bromelain has a significant impact on the thickness of skin affected by scleroderma. The current study results showed a significant reduction in dermal thickness and a considerable increase in epidermis thickness in all areas where bromelain was administered (Table 1). Since collagen is present in the dermal region and our drug formulation is designed to degrade collagen, it seems that this decrease in thickness might be attributed to collagen degradation in the dermis.

A study performed in 1964 reported the beneficial effects of bromelain therapy on improving the extremities' mobility and food intake of a scleroderma patient [7, 49]. Fibroblasts trans‐differentiation into myofibroblasts is the main feature of the fibrotic process in SSc [50]. Aichele et al. discovered that in vitro application of bromelain may diminish fibroblasts proliferation, migration and myofibroblasts differentiation [51]. Furthermore, topical administration of bromelain had favourable effects on necrotic tissue debridement, burn and surgical wound healing, and re‐epithelialisation (due to the presence of escharase component in bromelain) as well as decreased pain, erythema and oedema in chronic and surgical wounds [48, 52, 53, 54].

Previous investigations revealed the beneficial effects of bromelain in conditions linked with inflammation, such as rheumatoid arthritis, inflammatory bowel disease, severe coronavirus 2 acute respiratory syndrome (SARS‐CoV‐2), skin wounds and burns [21, 22, 55, 56] Since bromelain shows anti‐inflammatory properties by regulating bradykinin blood levels, inhibiting pro‐inflammatory factors (like prostaglandin E2 and thromboxane A2) and increasing anti‐inflammatory factors (including prostaglandin (PG) I2 and PGE1) [21, 22, 56].

Although there were no adverse effects detected in our study, gastrointestinal manifestations (like nausea, vomiting, diarrhoea and digestive problems), allergic reactions (such as rash, swelling, itching and breathing problems), headache, dizziness and palpitation were reported following bromelain administration route [57, 58]. Investigations evaluating bromelain toxicity indicated a lethal dose of LD_50_ greater than 10 g/kg in mice with no teratogenic or carcinogenic effects at dosages of 1.5 g/kg/day [59]. Accordingly, bromelain is considered safe, and clinical trials reported no severe side effects till the present time [22, 57].

In addition to clinical funding, such as skin softness and increased skin elasticity, our hypothesis regarding collagen degradation is supported. The subjective experience of the participant in this study further supports the effectiveness of our treatment, with significant improvements in skin softness and brightness reported just 10 days after starting the treatment. Additionally, the participant experienced reduced pain during blood sampling and found it easier to locate a vein.

Recent studies on niosomal drug delivery systems have demonstrated their potential in enhancing drug penetration, stability and therapeutic efficacy in dermatological conditions [37, 38]. The thin film hydration method used in this study aligns with previous research on cyclosporine and pentoxifylline niosomes, where optimised formulations improved drug encapsulation efficiency and skin retention [39, 40]. Additionally, embedding niosomes into a hydrogel matrix has been shown to increase localised drug deposition and controlled release, a strategy proven effective for topical psoriasis treatments [41]. The significant reduction in dermal thickness observed in our study further supports the hypothesis that niosomal encapsulation enhances bromelain's ability to degrade excess collagen, distinguishing this approach from conventional treatments that primarily inhibit collagen synthesis rather than actively reverse fibrosis. These findings reinforce the potential of niosomal hydrogels as a promising topical therapy for scleroderma‐related skin fibrosis.

The niosomal hydrogel provides an effective delivery system for bromelain, as the niosomes can penetrate the skin, resulting in collagen decomposition effectively. The high drug EE of 52.2% achieved in this study can be considered a favourable result compared to previous studies. For example, the loading capacity of bromelain in niosomes reported 34.89% [60]. It is worth noting that the loading of water‐soluble drugs in vesicular nanocarriers is typically low and does not exceed 10%–20% [60, 61]. In addition to the high drug loading, the monodispersity and small size of the niosomes, as confirmed by SEM imaging, indicate their potential for successful application in clinical applications, making them a promising candidates for clinical applications.

To the best of our knowledge, this is the first study that evaluated the effects of bromelain‐contained niosomal hydrogel on skin fibrosis of systemic sclerosis patients. Yet, there are limitations in the present study, including a small sample size and limited treatment duration. Also, the depth of penetration of the bromelain hydrogel was not measured. Therefore, further long‐term investigations with a large population are required to assess the safety and efficacy of niosomal bromelain in SSc skin involvement.

## 5 Conclusion

In conclusion, our study provides evidence for the potential use of niosomal hydrogel containing bromelain as an effective treatment for attenuating skin fibrosis in cases of scleroderma. Further studies with larger sample sizes and longer treatment durations are warranted to confirm these findings and evaluate the long‐term safety and efficacy of this treatment approach.

## Author Contributions

**Hanieh Ardeshiri:** conceptualization (lead), data curation (equal), investigation (equal), visualization (equal), writing – original draft (equal), writing – review and editing (equal). **Mina Ghorbani:** writing – original draft (equal), writing – review and editing (equal). **Mohammad Ali Nazarinia:** project administration (equal), supervision (equal), validation (equal). **Kimia Falamarzi:** writing – original draft (equal), writing – review and editing (equal). **Ali Mohammad Tamaddon:** methodology (equal), project administration (equal), supervision (equal), validation (equal). **Negar Azarpira:** project administration (equal), supervision (lead), validation (equal).

## Ethics Statement

Current study protocols were approved by the Ethics Committee of Shiraz University of Medical Sciences (Approval code: IR.SUMS.REC.1400.870) and written informed consent was obtained from the participant before enrolment in the study.

## Consent

The authors have nothing to report.

## Conflicts of Interest

The authors declare no conflicts of interest.

## Data Availability

The data that support the findings of this study are available from the corresponding author upon reasonable request.
